# The Relationship Between Academic Encouragement and Academic Self-Efficacy: A Moderated Mediation Model

**DOI:** 10.3389/fpsyg.2022.644243

**Published:** 2022-07-07

**Authors:** Zhun Gong, Xinian Jiao, Xinlei Xia, Haixin Yu, Cixian Lv

**Affiliations:** Normal College, Qingdao University, Qingdao, China

**Keywords:** encouragement, academic self-efficacy, campus connectedness, hope, academic engagement

## Abstract

To explore the influence mechanism and boundary conditions of academic encouragement on college students’ academic self-efficacy, this study did a questionnaire survey and used the four scales, namely, Academic Encouragement Scale (AES), Course Subscale of the College Self-Efficacy Inventory (CCSI), Adult Hope Scale (AHS), and Campus Connectedness Scale (CCS). The questionnaires were distributed both online and offline. A total of 355 questionnaires were distributed, with 267 valid returns. Among them, 139 were women (52.1%) and 128 were men (47.9%), and the age range is 18–24 years old. As for the grade level, 123 were first-year college students (46.1%), 58 were second-year college students (21.7%), and 86 were third-year college students (32.2%). The results of this study showed the following. (1) Campus connectedness or hope mediated the relations between (challenge-focused or potential-focused) encouragement and academic self-efficacy. (2) Academic engagement could not moderate the above mediation models.

## Introduction

Adler (1956) first suggested that encouragement is a central element of human development and psychotherapy. He believes that the experience of receiving encouragement is beneficial for individuals to regain their interest in society.

Dinkmeyer and Losoncy (1996) emphasized that encouragement was the process of facilitating the development of inner resources and courage in the individual toward positive movement. Nikelly and Dinkmeyer (1971) defined encouragement as a non-verbal attitude that communicates esteem and worth to an individual. Evans et al. (1997) proposed four dimensions of encouragement: (a) a positive view of oneself, (b) a positive view of others, (c) being open to experiences, and (d) a sense of belonging to others (Phelps et al., 2001). Later, some scholars argued that Adler’s definition lacked conceptual meaning. The conceptualization of encouragement includes dimensions such as being open to experiences and the courage to be imperfect (Dagley et al., 1999; Phelps et al., 2001). The conceptualization differed from the traditional mechanism of encouragement in psychology (Beets et al., 2010), which focuses on encouragement as an interpersonal act of social support. Wong defined encouragement as “the expression of affirmation through language or other symbolic representations to instill courage, perseverance, confidence, inspiration, or hope in a person” (Wong, 2015). On this basis, Wong et al. (2019a) introduced the concept of academic encouragement as the application of encouragement in academic scenarios, and further divided academic encouragement into challenge-focused encouragement and potential-focused encouragement, and developed the Academic Encouragement Scale (AES) to assess the two-factor structure of academic encouragement. In particular, challenge-focused encouragement focuses on the difficult situations that individuals face, while potential-focused encouragement focuses on the potential that individuals possess. While challenge-focused encouragement starts with helping individuals cope with current adversity, potential-focused encouragement starts with helping individuals understand their own value.

Previous studies have found a correlation between academic encouragement and academic self-efficacy (Byars-Winston et al., 2017; Won et al., 2017). In academic settings, encouragement may be more useful to students. Students who are encouraged may increase their academic performance and motivation (Guéguen et al., 2015; Alcott, 2017). A study has found that academic encouragement from others is beneficial in enhancing individuals’ social connections to improve their self-efficacy (Wong et al., 2019). Some college students reported that academic encouragement from friends and classmates helped to increase their sense of belonging to school (Glaser and Bingham, 2012) due to the fact that college students who experience academic encouragement from others may feel appreciated and cared for by others, which may facilitate their academic communication and enhance social connections.

Furthermore, academic encouragement may increase academic self-efficacy through cognitive pathways due to the fact that encouragement conveys a message of affirmation that can increase the recipient’s motivation and bring them hope (Main and Boughner, 2011). A study has found that college students’ experiences of receiving encouragement are positively correlated with hope (Khan, 2013) and that hope facilitates increased self-efficacy (Feldman and Kubota, 2015); therefore, students who have received academic encouragement may feel more confident in their ability to pursue their academic goals. Moreover, it was found that academic engagement was correlated with academic self-efficacy (Oriol-Granado et al., 2017), and that individuals’ academic self-efficacy was more likely to be motivated when academic engagement was higher. Therefore, academic engagement should be included in the study as a moderating variable.

**FIGURE 1:**
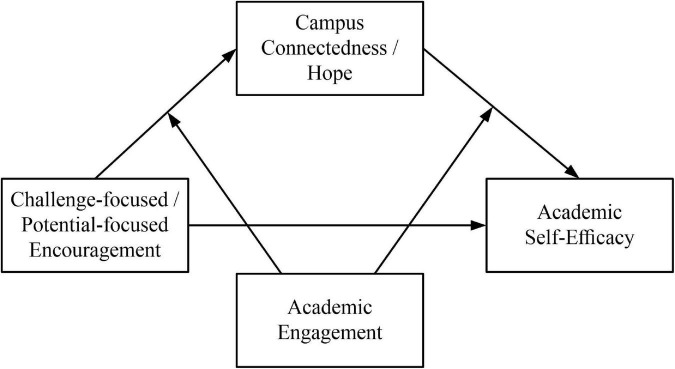
Theoretical model diagram.

In this context, we propose the following hypotheses, and the theoretical model diagram is shown in Figure 1.

H1: Campus connectedness can mediate the relation between challenge-focused encouragement and academic self-efficacy.

H2: Campus connectedness can mediate the relation between potential-focused encouragement and academic self-efficacy.

H3: Hope can mediate the relation between challenge-focused encouragement and academic self-efficacy.

H4: Hope can mediate the relation between potential-focused encouragement and academic self-efficacy.

H5: Academic engagement can moderate the above mediation models. Specifically, under conditions of higher academic engagement, the above relations are stronger.

## Materials and Methods

### Sample

A total of 355 questionnaires were distributed in this study. The valid return of questionnaires was 267, and the valid return rate was 75.2%. Among participants, 139 were women (52.1%) and 128 were men (47.9%), and the age range is 18–24 years old. As for the grade level, 123 were first-year college students (46.1%), 58 were second-year college students (21.7%), and 86 were third-year college students (32.2%). All participants were undergraduate students from universities in Qingdao who filled out the questionnaire online (most of them use the way to fill in the code on site, and a few of them use the way to fill in the WeChat forwarding).

### Measures

#### Academic Encouragement

This study used the AES developed by Wong et al. (2019b) for the assessment of academic encouragement. The scale has 10 items, and the scale is divided into two subscales: the challenge-focused encouragement scale (AES-challenge, 5 items) and the potential-focused encouragement scale (AES-potential, 5 items). The example item is “When I doubted my ability to learn, the people I respected encouraged me to believe in myself.” A 6-point Likert scale was used (1 = Strongly disagree; 6 = Strongly agree). Higher scores on the AES represent more effective encouragement from the person the participant respects. In this study, Cronbach’s alpha coefficient for the AES-Challenge scale was 0.92, and Cronbach’s alpha coefficient for AES-Potential was 0.90. Therefore, the scale had good reliability.

#### Academic Self-Efficacy

This study used the course subscale of the College Self-Efficacy Inventory (CSEI) developed by Solberg et al. (1993) for the assessment of academic self-efficacy. A total of seven questions were asked, using a 10-point Likert scale (1 = Not confident; 10 = Very confident), to rate the level of confidence in three aspects of college academics (including essay writing, classroom performance, and time management). The example item is “Complete assignments on time.” Higher scores on this scale represented higher academic self-efficacy of the participants. In the present study, Cronbach’s alpha coefficient for the scale was 0.90. Therefore, the scale had good reliability.

#### Hope

The adult hope scale (AHS) developed by Snyder et al. (1991) was used in this study. The scale has 8 items and uses a 4-point Likert scale (1 = Strongly disagree; 4 = Strongly agree). The example item is “Even when others are discouraged, I know I can find a way to solve the problem.” In the present study, Cronbach’s alpha coefficient for the scale was 0.88. Therefore, the scale had good reliability.

#### Campus Connectedness

The Campus Connectedness Scale (CCS), developed by Lee and Robbins (1995) and revised by Lee et al. (2002), was used in this study. The scale consists of 14 items. A six-point Likert scale was used (1 = Strongly disagree; 6 = Strongly agree), where items 2, 5, 6, 8, 10, 11, 13, and 14 were reverse-scored items. The example item is “I feel I can share my personal concerns with other students.” Higher scores on this scale represent higher levels of campus connectedness. In the present study, Cronbach’s alpha coefficient for the scale was 0.86. Therefore, the scale had good reliability.

#### Academic Engagement

This study referred to Crockett et al. (1987) and Dornbusch et al. (1987) and set an item asking participants to assess their level of academic engagement. A five-point Likert scale was used (1 = Very low, 5 = Very high), with higher scores on this item representing higher levels of academic engagement.

## Statistical Method

SPSS 25.0 and Hayes (2013) PROCESS v3.3 were used for the data input and analysis in this study. SPSS 25.0 was used for the reliability test, validity test, common method bias test, and correlation analysis. Additionally, PROCESS v.3.3 was used to test the moderated mediation model. To study the reliability and validity of the scales used in this study in the Chinese context, we have performed the reliability and validity tests. Since all the data collected in this study used self-assessment scales, in order to test for the common method bias, we referred to Podsakoff et al. (2003) to conduct a common method bias test. To study the correlations between variables, we have performed a correlation analysis. Finally, to test the hypotheses, we have performed a moderated mediation model test.

## Results

### Validity Test

We did the Kaiser-Meyer-Olkin (KMO) and Bartlett’s tests. The KMO value was 0.896, and Bartlett’s test reached a significant level (*p* < 0.001). This indicates that the scales used in this study have good validity.

### Common Method Bias Test

In this study, the common method test used Harman’s one-way method; the variance explained by the unrotated first factor was only 28.675%, which is much lower than the critical value of 40%. This indicates that the relationship between variables in this study is not affected by the common method bias.

### Correlation Analysis

The correlation coefficients between the variables are shown in Table 1.

**TABLE 1:** Correlation analysis.

Variable	*M (SD)*	1.	2.	3.	4.	5.
1. Challenge	21.46 (5.354)	1				
2. Potential	20.27 (5.599)	0.802**	1			
3. Campus Connectedness	62.15 (10.709)	0.310**	0.332**	1		
4. Hope	22.76 (4.257)	0.285**	0.389**	0.423**	1	
5. Academic Self-Efficacy	46.85 (11.933)	0.429**	0.430**	0.293**	0.465**	1

Challenge = challenge-focused encouragement, Potential = potential-focused encouragement, **p < 0.01.

### Hypothesis Test

We used Model 58 in Hayes (2013) PROCESS v3.3 to examine the moderated mediation mode. PROCESS examines the mediation and moderated effects by using a bias-corrected bootstrapping within a regression-based framework using an ordinary least squares estimator. Results are shown in Table 2.

**TABLE 2:** Hypothesis test.

Model 1	Campus connectedness			Academic self-efficacy		
	β	se	*t*	β	se	*t*
Constant	−0.007	0.627	−0.011	46.832	0.644	72.713**
Challenge	0.619	0.117	5.268**	0.819	0.127	6.458**
Academic engagement	0.216	0.437	0.494	1.207	0.448	2.691**
Challenge × Academic engagement	0.016	0.076	0.205			
Campus connectedness				0.193	0.063	3.045**
Campus connectedness × Academic engagement				0.032	0.04	0.782
*R* ^2^	0.097			0.235		
F	9.453**			20.166**		

**Model 2**	**Campus connectedness**			**Academic self-efficacy**		
	**β**	**se**	***t***	**β**	**se**	***t***

Constant	−0.032	0.623	−0.051	46.829	0.646	72.479**
Potential	0.642	0.113	5.69**	0.775	0.123	6.308**
Academic engagement	0.136	0.434	0.314	1.119	0.451	2.483*
Potential × Academic engagement	0.046	0.072	0.63			
Campus connectedness				0.186	0.064	2.911**
Campus connectedness × Academic engagement				0.035	0.04	0.871
*R* ^2^	0.112			0.231		
F	11.010**			19.626**		

**Model 3**	**Hope**			**Academic self-efficacy**		
	**β**	**se**	***t***	**β**	**se**	***t***

Constant	0.016	0.251	0.063	46.855	0.600	78.045**
Challenge	0.225	0.047	4.780**	0.701	0.118	5.952**
Academic engagement	−0.008	0.175	−0.045	1.241	0.418	2.968**
Challenge × Academic engagement	−0.037	0.031	−1.203			
Hope				1.045	0.147	7.094**
Hope × Academic engagement				−0.006	0.101	−0.063
*R* ^2^	0.086			0.335		
F	8.259**			32.933**		

**Model 4**	**Hope**			**Academic self-efficacy**		
	**β**	**se**	***t***	**β**	**se**	***t***

Constant	0.006	0.242	0.023	46.856	0.611	76.632**
Potential	0.295	0.044	6.742**	0.595	0.12	4.974**
Academic engagement	−0.052	0.169	−0.307	1.189	0.427	2.785**
Potential × Academic engagement	−0.008	0.028	−0.283			
Hope				0.992	0.156	6.347**
Hope × Academic engagement				−0.018	0.103	−0.176
*R* ^2^	0.152			0.310		
F	15.731**			29.398**		

Challenge = challenge-focused encouragement, Potential = potential-focused encouragement, *p < 0.05, **p < 0.01.

In Model 1, challenged-focused encouragement had a significant predictive effect on campus connectedness (*p* < 0.01). Campus connectedness had a significant predictive effect on academic self-efficacy (*p* < 0.01). Challenged-focused encouragement had a significant direct effect on academic self-efficacy as well (*p* < 0.01). Thus, campus connection played a partially mediating role between challenged-focused encouragement and academic self-efficacy. In summary, Hypothesis 1 was accepted.

In Model 2, potential-focused encouragement had a significant predictive effect on hope (*p* < 0.01). Hope had a significant predictive effect on academic self-efficacy (*p* < 0.01). Potential-focused encouragement had a significant direct effect on academic self-efficacy as well (*p* < 0.01). Thus, campus connection played a partially mediating role between potential-focused encouragement and academic self-efficacy. In summary, Hypothesis 2 was accepted.

In Model 3, challenged-focused encouragement had a significant predictive effect on hope (*p* < 0.01). Hope had a significant predictive effect on academic self-efficacy (*p* < 0.01). Challenged-focused encouragement had a significant direct effect on academic self-efficacy as well (*p* < 0.01). Thus, hope played a partially mediating role between challenged-focused encouragement and academic self-efficacy. In summary, Hypothesis 3 was accepted.

In Model 4, potential-focused encouragement had a significant predictive effect on hope (*p* < 0.01). Hope had a significant predictive effect on academic self-efficacy (*p* < 0.01). Potential-focused encouragement had a significant direct effect on academic self-efficacy as well (*p* < 0.01). Thus, hope played a partially mediating role between potential-focused encouragement and academic self-efficacy. In summary, Hypothesis 4 was accepted.

In the above models, none of the interaction terms of academic engagement was significant; therefore, the moderating effects of academic engagement on the above-mediated models were not significant. Therefore, Hypothesis 2 was rejected.

## Discussion

This study explored the relationship between academic encouragement and academic self-efficacy and further explored the mediating role played by campus connectedness and hope, as well as the moderating role of academic engagement. Through data analysis, we found that consistent with previous studies (Khan, 2013; Byars-Winston et al., 2017; Won et al., 2017; Wong et al., 2019a), students’ experiences of being encouraged in academics positively predicted their levels of academic self-efficacy.

Also, we also found a significant mediating role of campus connectedness and hope between academic encouragement and academic self-efficacy; therefore, Hypotheses 1–4 were accepted. Thus, encouragement may affect students’ academic self-efficacy through two pathways. One pathway is that students who have received academic encouragement may be motivated to connect with people on campus so that their sense of belonging on campus increases (Hamm and Faircloth, 2005; Glaser and Bingham, 2012), which in turn increases their academic self-efficacy. Another pathway is that students who have received academic encouragement may have more confidence in the goals they are pursuing (Main and Boughner, 2011), and their level of hope increases, which in turn increases their academic self-efficacy. Moreover, this study found that both academic encouragements that focus on challenge and encouragement that focuses on potential may lead to positive psychosocial outcomes for students. Therefore, school teachers and administrators should give more academic encouragement to students to help raise their level of campus connectedness and hope to further enhance their academic self-efficacy, which is conducive to their physical and mental development and academic progress.

In addition, this study found that the moderating effect of academic engagement between academic encouragement and academic self-efficacy was not significant, and therefore Hypothesis 5 was rejected. However, the main effect of academic engagement on academic self-efficacy was significant, suggesting that academic engagement is not a boundary condition for the relationship between academic encouragement and academic self-efficacy, but an independent variable affecting academic self-efficacy. Specifically, the higher the level of students’ academic engagement, the higher their level of academic self-efficacy (Oriol-Granado et al., 2017). At present, the mechanism of the influence of academic engagement on academic self-efficacy is unclear, and subsequent studies can explore the mechanism of the influence of academic engagement on academic self-efficacy research.

Since the samples in this study were all from China, which has geographical limitations, future studies can try cross-cultural studies to test whether the mechanisms and boundaries of the effects of academic encouragement on self-efficacy are consistent across cultures. This study used a combination of offline and online data collection, and since most of the data came from offline (74.91%), and there may be differences between online and offline data, in subsequent studies, more online data can be collected to test whether the results are consistent. Additionally, since this study used a cross-sectional study to validate the short-term effects of academic encouragement on academic self-efficacy, future research could attempt a follow-up study to test whether academic encouragement has long-term benefits on the increase of self-efficacy. Finally, the measurement instruments used in this study were all self-assessment scales and therefore may be biased, so follow-up studies could develop more objective measurement instruments.

## Conclusion

This study explored the mechanisms and boundary conditions of the effect of academic encouragement on academic self-efficacy and found that campus connections and hope were important mediating variables that influenced the relationship. Also, no boundary conditions had been found in this study for the relation between academic encouragement and academic self-efficacy, i.e., the relation was stable regardless of the level of academic engagement. This study revealed that in college education, teachers encouraging students while increasing their level of campus connections and hope can contribute to their academic self-efficacy.

## Data Availability Statement

The raw data supporting the conclusions of this article will be made available by the authors, without undue reservation.

## Ethics Statement

The studies involving human participants were reviewed and approved by the Institutional Review Board, Normal College, Qingdao University. The patients/participants provided their written informed consent to participate in this study.

## Author Contributions

ZG and XX designed, performed, analyzed the research, and wrote the research. ZG and HY critically reviewed and edited the manuscript. X-NJ and C-XL made substantial revisions. All authors contributed to the article and approved the submitted version.

## Conflict of Interest

The authors declare that the research was conducted in the absence of any commercial or financial relationships that could be construed as a potential conflict of interest.

## Publisher’s Note

All claims expressed in this article are solely those of the authors and do not necessarily represent those of their affiliated organizations, or those of the publisher, the editors and the reviewers. Any product that may be evaluated in this article, or claim that may be made by its manufacturer, is not guaranteed or endorsed by the publisher.
